# Human Papillomavirus (HPV) 16 E6 Variants in Tonsillar Cancer in Comparison to Those in Cervical Cancer in Stockholm, Sweden

**DOI:** 10.1371/journal.pone.0036239

**Published:** 2012-04-27

**Authors:** Juan Du, Cecilia Nordfors, Anders Näsman, Michal Sobkowiak, Mircea Romanitan, Tina Dalianis, Torbjörn Ramqvist

**Affiliations:** Department of Oncology-Pathology, Karolinska Institutet, Stockholm, Sweden; IPO, Inst Port Oncology, Portugal

## Abstract

**Background:**

Human papillomavirus (HPV), especially HPV16, is associated with the development of both cervical and tonsillar cancer and intratype variants in the amino acid sequence of the HPV16 E6 oncoprotein have been demonstrated to be associated with viral persistence and cancer lesions. For this reason the presence of HPV16 E6 variants in tonsillar squamous cell carcinoma (TSCC) in cervical cancer (CC), as well as in cervical samples (CS), were explored.

**Methods:**

HPV16 E6 was sequenced in 108 TSCC and 52 CC samples from patients diagnosed 2000–2008 in the County of Stockholm, and in 51 CS from young women attending a youth health center in Stockholm.

**Results:**

The rare E6 variant R10G was relatively frequent (19%) in TSCC, absent in CC and infrequent (4%) in CS, while the well-known L83V variant was common in TSCC (40%), CC (31%), and CS (29%). The difference for R10G was significant between TSCC and CC (p = 0.0003), as well as between TSCC and CS (p = 0.009). The HPV16 European phylogenetic lineage and its derivatives dominated in all samples (>90%).

**Conclusion:**

The relatively high frequency of the R10G variant in TSCC, as compared to what has been found in CC both in the present study as well as in several other studies in different countries, may indicate a difference between TSCC and CC with regard to tumor induction and development. Alternatively, there could be differences with regard to the oral and cervical prevalence of this variant that need to be explored further.

## Introduction

Infection with high-risk (HR) human papillomaviruses (HPV) has since the beginning of 1980s been recognized to cause the development of cervical cancer and more recently also oropharyngeal cancer [1], [2]. Among oropharyngeal cancers mainly tonsillar and base of tongue cancers are associated with HPV. Notably, the incidence of HPV positive oropharyngeal cancer has increased during the last decades, both in Europe and the US [3]–[5]. According to Swedish Cancer Registry, in Sweden, presently over 400 new cases of cervical cancer and around 200 cases of tonsillar cancer are diagnosed every year.

All HPV types known to be associated with oropharyngeal cancer are also linked to cervical cancer and HPV16 dominates in both, causing 50–60% of all HPV positive cervical cancer and roughly 90% of all HPV positive tonsillar cancer. In addition, while the HPV genome is mostly integrated in cervical cancer, it is often episomal in tonsillar cancer [6]. This indicates that, although there are many similarities between these two tumor types there could also be differences, both with regard to the potential of HPV16 to cause tumor development, and to the way different HPV types cause tumors.

Variants of the HPV16 genome in cervical cancer, in pre-stages of cervical cancer, and in cervical samples from different geographical areas have been studied extensively and HPV has been classified into phylogenetic lineages as European (E), Asian (As), Asian-American (AA), African-1 and -2 (Af-1 and Af-2), and North American1 (NA1) [7]–[11].

The major transforming proteins of HPV16, E6 and E7, target p53 and Rb respectively, inhibit apoptosis and promote proliferation and therefore most studies on HPV16 variants have focused on E6 and E7 in addition to the studies of the non-coding control region (NCCR). Variants have been shown to often differ in E6 and NCCR, while the E7 protein is often more conserved [12], [13]. In addition, to the major variant lineages, variants with single nucleotide alterations are frequently found, especially in tumors. The most frequent of these mutations is the T to G transition at nt 350 in E6, abbreviated as E-T350G, causing an amino acid change from leucine to valanine at a.a. 83 (L83V). Several studies on the L83V variant have demonstrated an association with viral persistence and cancer progression of cervical carcinoma, although some later studies have failed to confirm this association [14]–[17]. Other variants have also been linked to properties important for tumor development such as e.g. those with the ability to inhibit p53 transactivation [18].

While many studies on HPV16 variants have been performed in cervical cancer, very few studies of this kind have been made in head neck cancer [19], [20]. The purpose of this study was to therefore to examine if there were any differences in occurrence and frequency of HPV16 E6 variants between tonsillar cancer and cervical cancer. For this reason the prevalence of HPV16 E6 sequences were compared in cervical and tonsillar cancer samples from patients diagnosed during the same time frame in the County of Stockholm, Sweden. In addition, to compare the data obtained from these tumors with the frequencies of HPV16 variants in healthy persons, HPV16 E6 was also analyzed in cervical samples from healthy young women at a youth clinic in Stockholm.

## Results

### HPV16 E6 variants at amino acid R10G and L83V

Fifty-five HPV16 positive TSSC, 52 CC and 51 CS were analyzed with regard to HPV16 E6 sequence, Table S1. The most remarkable difference between TSCC and CC was found with regard to the frequencies of the E-A131G variant causing a change from arginine to glycine in a.a. 10 (R10G). This mutation was present in 22% (12/55) of TSCC, while completely lacking in the CC samples and only present in 4% (2/51) of cervical samples. This difference was significant both between TSCC and CC (p = 0.0003) as well as between TSCC and CS (p = 0.009).

The E-T350G variant was the most common one among all the three groups of samples. This variant, causing a shift from leucine to valine in aa 83 (L83V), was especially common in TSCC (45%) followed by CC (31%) and CS (29%) respectively.

### Validation of R10G and L83V frequencies in further TSCC samples

To further validate the increased frequencies of R10G and L83V mutations in TSCC, 53 additional TSCC samples, from patients diagnosed at the same hospital during the same period, were evaluated only for the R10G and L83V mutations. Among these 53 samples 17% (9/53) carried the R10G and 34% (18/53) the L83V mutation. When all the 108 samples were summarized 19% had R10G and 40% the L83V mutation. The majority (12/21) of the E6 from TSCC harboring R10G also contained L83V. No significant difference was observed between the originally selected 55 TSCC and the extended TCCS group with regard to these two mutations.

### Frequency of other variants

Mutations at other sites were less common for all types of samples, and only 19/28 of these affected the amino acid sequence. The most common were Q14H and H78Y, both present in 4% of CC and CS, while 7% of TSCC had Q14H and H78Y. In addition, two cervical cancer samples had 27 bp repeats between nt 528 and 554.

### Distribution of different phylogenetic lineages

As detailed in Table S1, the HPV16 E6 European prototype was found in 38% of TSCC, 65% of CC and 59% of CS. However, when samples with the European type containing minor nucleotide differences (including R10G and L83V) were included, these figures increased to 93, 94 and 96% respectively. As also shown in the Table S1 other types, African-1 and 2, East Asian. Asian American and North American were only found in few samples.

### Correlation of HPV16 E6 variants in tonsillar cancer with clinical parameters

The TSCC samples were compared with regard to patient and tumor characteristics based on the presence of R10G, L83V or absence of these variants as presented in Table 1. There were however no significant differences for any of these parameters, although there was a tendency for tumors with R10G to have a lower T stage.

**Table 1:** Patient and tumor characteristics for TSCC.

Patient and tumor characteristics	All patients (n = 108)		Patients with R10G (n = 21)		Patients without R10G (n = 87)		Patients with L83V (n = 43)		Patients without L83V (n = 65)	
Gender	n	%	n	%	n	%	n	%	n	%
Male	84	78%	17	81%	67	77%	32	74%	52	80%
Female	24	22%	4	19%	20	23%	11	26%	13	20%
Mean age	59.7		59.4		59.8		60.1		59.8	
**TNM classification**										
T1	24	22%	6	29%	18	21%	10	23%	14	22%
T2	44	41%	10	48%	34	39%	17	40%	27	42%
T3	24	22%	3	14%	21	24%	9	21%	15	23%
T4a-b	16	15%	2	10%	14	16%	7	16%	9	14%
N0	17	16%	4	19%	13	15%	5	12%	12	18%
N1	26	24%	4	19%	22	25%	14	33%	12	18%
N2a-c	63	58%	14	67%	49	56%	24	56%	39	60%
N3	3	3%	0	0%	3	3%	1	2%	2	3%
M0	106	98%	21	100%	85	98%	42	98%	64	98%
M1	2	2%	0	0%	2	2%	1	2%	1	2%
**Stage**										
I	2	2%	1	5%	1	1%	2	5%	0	0%
II	7	6%	2	10%	5	6%	2	5%	5	8%
III	28	26%	5	24%	23	26%	11	26%	17	26%
IV	72	67%	14	67%	58	67%	29	67%	43	66%

The presence of the R10G or L83V variants in tonsillar cancer was analyzed in correlation to the 3-year disease-free survival (DSF) of the patients. However, no significant correlation was found when DSF was compared between patients with presence or absence of the R10G variant or between patients with presence or absence of the L83V variant (data not shown).

## Discussion

In the present study, we have analyzed and compared the frequency of different HPV16 E6 variants in TSCC, CC and CS, all obtained from the Stockholm area during the same time period. We found that HPV16 R10G was relatively common in TSCC, absent in CC and rare in CS. The HPV 16 European lineage was dominating and the L83V variant was very common in all three categories of samples.

The fact that the otherwise rare HPV16 E6 variant R10G was observed in TSCC, but not in CC in patients diagnosed at the same time period from the same hospital is striking. This variant has to our knowledge always been rare, although observed in CC in some other studies [11], [13], [18]. Though the time from HPV infection to the development of tonsillar cancer is unknown it is likely similar between cervical and tonsillar cancer, since the median age of the patients is comparable. Thus, it is reasonable to assume that the patients in this study with cervical and tonsillar cancer were most likely infected with HPV16 during approximately the same period. The reason for the high frequency of R10G variant in TSCC from Stockholm, as compared to CC from both Stockholm and other areas, is unknown. One possibility is that this HPV16 variant for some reason was/is more common in the oral as compared to the genital area. Further studies on HPV16 obtained from oral samples are needed to explore this possibility. Another possibility could be that this variant of HPV16 has a higher propensity to cause TSCC. Notably, although the majority of patients with TSCC were men, and only women have CC, there were no sex-related differences in the relative frequencies of R10G in TSCC. The collected data indicate that virtually all patients carrying the R10G variant had Sweden as their country of origin, however we do not have information if they have moved to Stockholm from other parts of Sweden.

It is well established that HPV16 E6 together with E6AP (E6-associated protein) forms a complex that specifically targets p53 and mediate p53 degradation, which is essential for tumorigenesis [21], [22]. Several studies have been performed on the R10G variant in cervical cancers. The R10G mutation is in the N-terminal end, important for p53 binding and degradation, and it has also been shown to result in a modified B/T cell epitope [13], [23], [24]. It has been demonstrated that both E6 with R10G and L83V retain the ability to abrogate growth arrest, and strongly reduce the steady state levels of p53 [25]. However, while E6 with R10G has a decreased binding to E6BP (E6-binding protein) and reduced ability to induce Bax degradation, E6 with L83V E6 has an increased binding to E6BP and retained ability to induce Bax degradation compared to prototype HPV16 E6 [18]. E6 with both the R10G and L83 alteration, which 9/12 TSCC had in this study, behaved similar to the prototype [18]. In addition, the R10G site has also been reported to be essential for nuclear localization signal [26]. Thus, several different features of the R10G site may influence cancer progression, even though it is unclear to what degree. In the present study no significant difference was observed with regard to the 3-year disease-free survival among the patients with TSCC carrying or not carrying HPV16 E6 R10G variant. This argues against the possibility that this variant would cause more aggressively growing TSCC.

Other variants of HPV16 E6, especially L83V, differ geographically and may affect the pathological risk [27]. In this study, L83V was the most common variant in all types of material, which is in line with many other observations both in the genital and head and neck region [16], [19], [20], [28], [29]. Here, a higher prevalence of L83V was found in TSCC compared to CC and CS, while the prevalence in the latter categories were roughly in agreement with previous reports[12], [14], [29]–[31]. There are however several studies demonstrating an association between L83V and increased virus persistence, and risk of cervical neoplasia and cancer progression [28], [32]. Furthermore, studies on HPV16 E6 variants and the cellular immune response indicate specific changes, that from a host-virus immunology point of view may explain a modified oncogenic potential [33]–[35]. Noteworthy, we did not find any significant correlation between the presence or absence of the L83V variant and the 3-year disease free survival of the patients.

It is noteworthy that the European HPV16 variant clearly has about the same very high frequency in CS from young women, obtained 2009–2010, as in CC samples. With the increase in travelling during the last decades an increase in HPV16 variants from other geographical areas would not have been unexpected, but no such increase was observed.

A limitation in the present study is the number of analyzed samples. Nevertheless, the 108 TSCC analyzed constitute approximately 65% of all patients from the County of Stockholm with HPV16 positive TSCC diagnosed during this time frame and where biopsies were available [1], [36]. Another limitation is that only the E6 region was analyzed. Thus, if there were any differences in the NCCR, E7 or E5 regions between the TSCC and CC samples, these would have been missed.

In this study we found a significant difference in the distribution of HPV16 variants between TSCC and CC samples from patients admitted to the same hospital during the specific time period. More specifically, the rare R10G variant was significantly more common in TSCC than in CC, and was frequently in combination with the L83V variant in the former tumors. Further studies are needed to investigate if the R10G variant may be more common in oral as compared to cervical samples. In addition, there was relative concordance between the presence of variants in CC and CS.

## Materials and Methods

### Ethics statement

The study was approved by the Ethical Committee at Karolinska Institutet, Stockholm, Sweden, according to the ethical permissions 2005/431-31/4, 2005/1330-32, 2008/870-31/4, 2008/813-31/2 and 2009/1278-31/4. Written informed consent was obtained from all participants in the study.

### Tumor and cervical samples material

**Figure 1:**
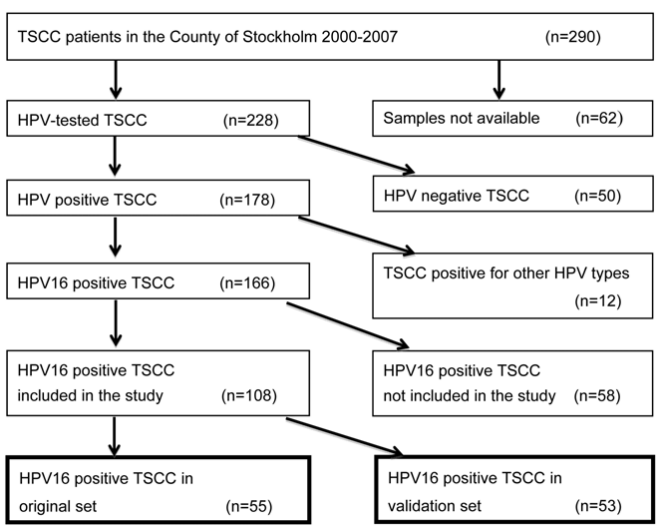
Flow chart showing the tumors from patients diagnosed with TSCC in the County of Stockholm 2000–2007 and the TSCC selected for inclusion in the study.

Pre-treatment paraffin embedded tumour biopsies from patients from the County of Stockholm, diagnosed 2000–2007, with tonsillar squamous cell carcinoma (TSCC) or 2003–2008 with cancer of the uterine cervix (CC). All tumor samples had previously been analyzed for the presence and type of HPV [1], [36]–[38]. Among the 166 TSCC and 90 CC that were HPV16 positive, 55 TSCC and 52 CC were randomly selected. In the second part of the study, for validation, 53 additional, randomly selected, HPV16 positive TSCC from the same time frame were included. A flow chart for the TSCC samples is presented in Figure 1. In addition, 51 HPV16 positive cervical samples (CS), from a previous study from 2009–2010 at a youth health center in Stockholm [39] were randomly selected and analyzed. The mean ages of the cancer patients were 59.7 y (median 59 y, range 30–84 y) for TSCC patients and 50.3 y (median 45 y, range 25–88 y) for CC patients while the mean age for the women at the youth clinic was 19.9 y (median 20 y, range16–22 y).

### Sequencing

DNA was extracted and analyzed for the presence of HPV and HPV type as described in previous studies [1], [37], [39]. For the analysis of HPV16 E6 from cancer tissues two primer pairs covering the whole E6 region was utilized. 5′- CCGGTTAGTATAAAAGCAGACAT-3′ together with 5′-TGCTGTTCTAATGTTGTTCC-3′ amplifying bp 57–375 and 5′- GGAATCCATATGCTGTATGT-3′ together with 5′- TGCAATGTAGGTGTATCTCC-3′ amplifying bp 273–587. For cervical samples one primer pair covering the whole of E6 was utilized; 5′-CCGGTTAGTATAAAAGCAGACAT-3′ and 5′-GTACCCTCTTCCCCATTGGT-3′ amplifying bp 57–902. PCR was performed with annealing temperature at 49°C and products were purified using ExoSAP-IT (USB,VWR) according to the protocol of the manufacturer. A PCR for sequencing was performed on the purified products with Big Dye™ terminator (Applied biosystems) and the amplicons were analyzed in an Applied Biosystems 3130 sequencer. Both DNA strands were sequenced and the DNA sequence and corresponding amino acid sequence was compared with the reference European HPV16 sequence (reference code: NC_001526.1) using the Sci-Ed software (Science and Educational Software, Durham, NC, USA). Three investigators (JD, CC and MS) were involved in the analysis of the sequences. For cases where the result obtained was unclear, the sequencing was repeated.

### Statistical analysis

Differences in frequencies of variants for TSCC and CC or CS were evaluated by chi-square and Fischer's exact test (two-tailed) using on-line statistical analysis software (GraphPad Software). Mean age was the age at the first diagnosis.

Disease-free survival (DFS) was defined as time from the date of diagnosis to the date of the last known occasion that the patient was disease-free, or the date of disease recurrence (local, regional or distant recurrence). Death without documented recurrence was censored at the date of death. Cumulative survival was calculated with the Kaplan-Meier method and analyzed with the log-rank test. Analysis of survival was performed in SPSS (IBM SPSS Statistics, version 20).

## Supporting Information

Table S1 The frequencies of different HPV16E6 variants in cervical cancer (CC), tonsillar squamous cell carcinoma (TSCC) and cervical samples (CS).
(DOC)
