# Supplementary material for: Human Papillomavirus (HPV) 16 E6 Variants in Tonsillar Cancer in Comparison to Those in Cervical Cancer in Stockholm, Sweden
Source: PLoS One. 2012 Apr 27;7(4):e36239. doi: 10.1371/journal.pone.0036239 (PMC3338690; doi:10.1371/journal.pone.0036239)
Supplement: Table S1 — The frequencies of different HPV16E6 variants in cervical cancer (CC), tonsillar squamous cell carcinoma (TSCC) and cervical samples (CS). (DOC) [file pone.0036239.s001.doc]

| Nucleotide position | | 1  0  9 | | 1  1  2 | 1  3  1 | 1  3  2 | 1  3  5 | 1  4  3 | 1  4  5 | 1  6  4 | 1  7  8 | 1  8  3 | 1  8  7 | 1  8  8 | 2  1  2 | 2  3  3 | 2  4  5 | 2  5  6 | 2  7  0 | 2  8  6 | 2  8  9 | 2  9  4 | 3  3  5 | 3  5  0 | 3  6  1 | 3  6  3 | 3  6  8 | 4  0  3 | 4  6  5 | 5  3  2 | CC  n=52 | | | TSCC  n=55 | | | CS  n=51 | | | |
| --- | --- | --- | --- | --- | --- | --- | --- | --- | --- | --- | --- | --- | --- | --- | --- | --- | --- | --- | --- | --- | --- | --- | --- | --- | --- | --- | --- | --- | --- | --- | --- | --- | --- | --- | --- | --- | --- | --- | --- | --- |
| Lineage^1^ | |  |  |  |  |  |  |  |  |  |  |  |  |  |  |  |  |  |  |  |  |  |  |  |  |  |  |  |  |  | n | % | | n | % | | n | | | % |
| E prototype | | T | | G | A | G | A | C | G | A | T | T | A | G | T | G | C | C | A | T | A | A | C | T | A | C | G | A | A | A | 34 | 65 | | 21 | 38 | | 30 | | | 59 |
| E | |  | |  | G^2^ |  |  |  |  |  |  |  |  |  |  |  |  |  |  |  |  |  |  |  |  |  |  |  |  |  | 0 | 0 | | 3 | 5 | | 0 | | | 0 |
| E G131 | |  | |  | G |  |  |  |  |  |  |  |  |  |  |  |  |  |  |  |  |  |  | G |  |  |  |  |  |  | 0 | 0 | | 9 | 16 | | 2 | | | 4 |
| E | |  | |  |  |  |  |  |  |  |  |  |  |  |  |  |  |  |  |  |  |  |  | G |  |  |  |  |  |  | 10 | 19 | | 8 | 15 | | 11 | | | 22 |
| E | |  | |  |  |  |  |  |  |  |  |  |  |  |  |  |  | t^2^ |  |  |  |  |  | G |  |  |  |  |  |  | 3 | 6 | | 2 | 4 | | 0 | | | 0 |
| E | | c | |  |  |  |  |  |  |  |  |  |  |  |  |  |  |  |  |  |  |  |  | G |  |  |  |  |  |  | 0 | 0 | | 1 | 2 | | 1 | | | 2 |
| E? | |  | | T |  |  |  |  |  |  |  |  |  |  |  |  |  |  |  |  |  |  |  | G |  |  |  |  |  |  | 0 | 0 | | 1 | 2 | | 0 | | | 0 |
| E? | |  | |  |  |  |  |  |  |  |  |  |  |  |  |  |  |  |  |  |  |  |  | G |  | G |  |  |  |  | 0 | 0 | | 1 | 2 | | 0 | | | 0 |
| E? | |  | |  |  |  |  |  |  |  |  |  |  | C |  | A |  |  |  |  |  |  |  | G |  |  |  |  |  |  | 1 | 2 | | 0 | 0 | | 0 | | | 0 |
| AA | |  | |  |  |  |  |  | T |  |  |  |  |  |  |  |  |  |  | a | g |  | T | G |  |  |  |  |  | g | 1 | 2 | | 2 | 4 | | 0 | | | 0 |
| AA | |  | |  |  |  |  |  | T |  |  | G |  |  |  |  |  |  |  | a | g |  | T | G |  |  |  |  |  | g | 0 | 0 | | 1 | 2 | | 0 | | | 0 |
| NA1 | |  | |  |  |  |  |  | T |  |  |  |  |  |  |  |  |  |  | a | g |  | T | G |  |  |  |  |  |  | 1 | 2 | | 0 | 0 | | 0 | | | 0 |
| Af-2 | | c | |  |  | T |  | G | T |  |  |  |  |  |  |  |  |  |  | a | g |  | T |  |  |  |  | g |  |  | 0 | 0 | | 1 | 2 | | 1 | | | 2 |
| Af-1 | |  | |  |  | C |  | G | T |  |  |  |  |  |  |  |  |  |  | a | g |  | T |  |  |  |  |  |  |  | 0 | 0 | | 0 | 0 | | 1 | | | 2 |
| E | |  | |  |  |  |  |  |  |  |  |  |  |  | c |  |  |  |  |  |  |  |  |  |  |  |  |  |  |  | 1 | 2 | | 0 | 0 | | 0 | | | 0 |
| As | |  | |  |  |  |  |  |  |  | G |  |  |  |  |  |  |  |  |  |  |  |  |  |  |  |  |  |  |  | 1 | 2 | | 0 | 0 | | 0 | | | 0 |
| E? | |  | |  |  |  |  |  |  |  |  |  |  |  |  | A |  |  |  |  |  |  |  |  |  |  |  |  |  |  | 0 | 0 | | 1 | 2 | | 0 | | | 0 |
| E? | |  | |  |  |  |  |  |  |  |  |  |  |  |  |  |  |  |  |  |  |  |  |  |  |  | C |  |  |  | 0 | 0 | | 1 | 2 | | 0 | | | 0 |
| E | |  | |  |  |  |  |  |  |  |  |  |  |  |  |  | T |  |  |  |  |  |  |  |  |  |  |  |  |  | 0 | 0 | | 1 | 2 | | 0 | | | 0 |
| E? | |  | |  |  |  |  |  |  |  |  |  |  |  |  |  |  |  | C |  |  |  |  |  |  |  |  |  | G |  | 0 | 0 | | 1 | 2 | | 0 | | | 0 |
| E | |  | |  |  |  | C |  |  |  |  |  |  |  |  |  |  |  |  |  |  |  |  |  |  |  |  |  |  |  | 0 | 0 | | 1 | 2 | | 0 | | | 0 |
| E? | |  | |  |  |  |  |  |  |  |  |  |  |  |  |  |  |  |  |  |  | C |  | G |  |  |  |  |  |  | 0 | 0 | | 0 | 0 | | 1 | | | 2 |
| E? | |  | |  |  |  |  |  |  |  |  |  |  |  |  |  |  |  |  |  |  |  |  |  | t |  |  |  |  |  | 0 | 0 | | 0 | 0 | | 1 | | | 2 |
| E | |  | |  |  |  |  |  |  |  |  |  | g |  |  |  |  |  |  |  |  |  |  |  |  |  |  |  |  |  | 0 | 0 | | 0 | 0 | | 2 | | | 4 |
| E? | |  | |  |  |  |  |  |  | T |  |  |  |  |  |  |  |  |  |  |  |  |  |  |  |  |  |  |  |  | 0 | 0 | | 0 | 0 | | 1 | | | 2 |
| a.a. no | | 2 | | 3 | 10 | 10 | 11 | 14 | 14 | 21 | 25 | 27 | 28 | 29 | 37 | 44 | 48 | 51 | 56 | 61 | 62 | 64 | 78 | 83 | 86 | 87 | 89 | 100 | 121 | 143 |  |  | |  |  | |  | | |  |
| a.a. in EU | |  | | Q | R | R | K | Q | Q | T | D | I |  | E |  | D | R |  | D |  |  | D | H | L |  | T | E |  | K |  |  |  | |  |  | |  | | |  |
| Variant aa | |  | | H | G | I/T | T | D^3^ | H^3^ | S | E | R |  | Q |  | N | W |  | A |  |  | A | Y | V |  | R | Q |  | R |  |  |  | |  |  | |  | | |  |
| CC  n=52 | n | | 0 | 0 | 0 | 0 | 0 | 0 | 2 | 0 | 1 | 0 | 0 | 1 | 1 | 1 | 0 | 3 | 0 | 2 | 2 | 0 | 2 | 16 | 0 | 0 | 0 | 0 | 0 | 1 |  | |  | | |  | |  |  | |
|  | % | | 0 | 0 | 0 | 0 | 0 | 0 | 4 | 0 | 2 | 0 | 0 | 2 | 2 | 2 | 0 | 6 | 0 | 4 | 4 | 0 | 4 | 31 | 0 | 0 | 0 | 0 | 0 | 2 |  | |  | | |  | |  |  | |
| TSCC  n=55 | n | | 2 | 1 | 12 | 1 | 1 | 1 | 4 | 0 | 0 | 1 | 0 | 0 | 0 | 1 | 1 | 2 | 1 | 4 | 4 | 0 | 4 | 25 | 0 | 1 | 1 | 1 | 1 | 3 |  | |  | | |  | |  |  | |
|  | % | | 4 | 2 | 22 | 2 | 2 | 2 | 7 | 0 | 0 | 2 | 0 | 0 | 0 | 2 | 2 | 4 | 2 | 7 | 7 | 0 | 7 | 45 | 0 | 2 | 2 | 2 | 2 | 5 |  | |  | | |  | |  |  | |
| CS  n=51 | n | | 2 | 0 | 2 | 2 | 0 | 2 | 2 | 1 | 0 | 0 | 2 | 0 | 0 | 0 | 0 | 0 | 0 | 2 | 2 | 1 | 2 | 15 | 1 | 0 | 0 | 1 | 0 | 0 |  | |  | | |  | |  |  | |
|  | % | | 4 | 0 | 4 | 4 | 0 | 4 | 4 | 2 | 0 | 0 | 4 | 0 | 0 | 0 | 0 | 0 | 0 | 4 | 4 | 2 | 4 | 29 | 2 | 0 | 0 | 2 | 0 | 0 |  | |  | | |  | |  |  | |

^1^Abbreviations for lineages: E, European; As, Asian; AA, Asian-American; Af-1 and Af-2, African-1 and -2; NA1, North American 1; E G131, European German 131; E? denotes probably European. (Huertas-Salgado et al., 2011; Terai et al., 2002 )

^2^Nucleotide alterations with capital letters affect the a.a. sequence while alterations with lower case letters do not.

^3^When nucleotides 143 and 145 are G and T respectively, a.a. 14 is D; when they are C and T respectively, a.a.14 is H.
